# Supplementary material for: Impact of Matrix Species and Mass Spectrometry on Matrix Effects in Multi-Residue Pesticide Analysis Based on QuEChERS-LC-MS
Source: Foods. 2023 Mar 13;12(6):1226. doi: 10.3390/foods12061226 (PMC10048110; doi:10.3390/foods12061226)
Supplement: Supplementary file 1 [file foods-12-01226-s001.zip › foods-2221992-supplementary.pdf]

Supplementary for: Impact of matrix species and mass spectrometries on matrix effects in multi-residue pesticide analysis based on QuEChERS-LC-MS

Shuang Zhang 1,2, Zhiyong He 1,2, Maomao Zeng 1,2, Chen Jie 1,2,*

[1] State Key Laboratory of Food Science and Technology, Jiangnan University, Wuxi, Jiangsu 214122, China
[2] International Joint Laboratory on Food Safety, Jiangnan University, Wuxi, Jiangsu 214122, China
[3] Joint Laboratory of Omics Technologies for Special Food, Jiangnan University, Wuxi, Jiangsu 214122, China
***** Correspondence: chenjie@jiangnan.edu.cn; Tel.: +86-139-6176-1357

Table S1. Chemical abstracts service number (CAS No.), chemical classes, usage types, Octanol-water partition coefficient (K_{ow}), retention time (t_R, min), and adduct as well as theoretical m/z of precursor ions for 73 pesticides.

**Name**	**CAS No.**	**Chemical classes**	**Usage types**	**K_{ow}**	**t_R (min)**	**Adduct**	**Theoretical m/z**
Alachlor	15972-60-8	Chloroacetamide	Herbicide	3.09	9.45	$[M+H]^+$	270.1255
Ametryn	834-12-8	Triazine	Herbicide	2.63	6.81	$[M+H]^+$	228.1277
Atrazine	1912-24-9	Triazine	Herbicide	2.7	7.61	$[M+H]^+$	216.1011
Carbendazim	10605-21-7	Benzimidazole	Fungicide, Metabolite	1.48	4.02	$[M+H]^+$	192.0768
Chlordimeform	6164-98-3	Formamidine	Acaricide, Insecticide, Ovicide	2.89	4.87	$[M+H]^+$	197.0804
Chlorpyrifos	2921-88-2	Organophosphate	Insecticide	4.7	11.16	$[M+H]^+$	321.9023
Chlorpyrifos-methyl	5598-13-0	Organophosphate	Insecticide, Acaricide	4	8.56	$[M+H]^+$	349.9336
Chlorsulfuron	64902-72-3	Sulfonylurea	Herbicide	2.14	7.33	$[M+H]^+$	358.0371
Coumaphos10.	56-72-4	Organophosphate	Insecticide	3.89	10.1	$[M+H]^+$	363.0217
Cyflufenamid	180409-60-3	Amidoxine	Fungicide	4.7	10.36	$[M+H]^+$	413.1283
DEET	134-62-3	Unclassified	Insecticide, Repellent	2.18	7.7	$[M+H]^+$	192.1383
Diazinon	333-41-5	Organophosphate	Insecticide, Acaricide, Repellent, Veterinary substance	3.69	10.19	$[M+H]^+$	305.1083
Dichlorvos	62-73-7	Organophosphate	Insecticide, Acaricide	1.9	6.87	$[M+H]^+$	220.9532
Diethofencarb	87130-20-9	Carbamate	Fungicide	2.89	8.64	$[M+H]^+$	268.1543
Difenoconazole	119446-68-3	Triazole	Fungicide	4.36	9.76	$[M+H]^+$	406.0720
Dimethenamid	87674-68-8	Chloroacetamide	Herbicide	2.2	8.79	$[M+H]^+$	276.0820
Dimethoate	60-51-5	Organophosphate	Insecticide, Acaricide, Metabolite	0.75	5.72	$[M+H]^+$	230.0069
Disulfoton	298-04-4	Organophosphate	Insecticide, Acaricide	3.95	7.3	/	/

Edifenphos	17109-49-8	Organophosphate	Fungicide	3.83	9.65	$[M+H]^+$	311.0324
Epoxiconazole	135319-73-2	Triazole	Fungicide	3.3	8.84	$[M+H]^+$	330.0804
Ethiofencarb	29973-13-5	Carbamate	Insecticide	2.04	7.67	$[M+H]^+$	226.0896
Ethoprophos	13194-48-4	Organophosphate	Insecticide, Nematicide	2.99	9.04	$[M+H]^+$	243.0637
Ethion	563-12-2	Organophosphate	Insecticide, Acaricide, Metabolite	5.07	10.28	$[M+H]^+$	384.9949
Fenarimol	60168-88-9	Pyrimidine	Fungicide	3.69	8.7	$[M+H]^+$	331.0399
Fenchlorphos	299-84-3	Organophosphate	Insecticide, Veterinary substance	4.88	8.56	$[M+H]^+$	320.9070
Fenitrothion	2255-17-6	Organophosphate	Insecticide	3.24	8.8	/	/
Fenobucarb	3766-81-2	Carbamate	Insecticide	2.78	8.5	/	/
Fenpropathrin	39515-41-8	Pyrethroid	Insecticide, Acaricide	6.04	11.19	$[M+H]^+$	350.1751
Fenthion	55-38-9	Organophosphate	Insecticide, Veterinary substance, Avicide	4.84	9.9	$[M+H]^+$	279.0273
Fluazifop-butyl	69806-50-4	Aryloxyphenoxy pr-opionate	Herbicide	4.5	9.34	$[M+H]^+$	384.1417
Fludioxonil	131341-86-1	Phenylpyrrole	Fungicide	4.12	8.5	$[M+H]^+$	249.0470
Fluquinconazole	136426-54-5	Triazole	Fungicide	3.24	8.96	$[M+H]^+$	376.0163
Flutriafol	76674-21-0	Triazole	Fungicide	2.31	7.41	$[M+H]^+$	302.1099
Hexythiazox	78587-05-0	Carboxamide	Acaricide	2.67	11.21	$[M+H]^+$	353.1085
Iprobenfos	26087-47-8	Organophosphate	Fungicide	3.37	7.62	$[M+H]^+$	289.1022
Isazofos	42509-80-8	Organophosphate	Insecticide, Nematicide	3.1	9.28	$[M+H]^+$	314.0490
Isoprocarb	2631-40-5	Carbamate	Insecticide	2.32	9.28	$[M+H]^+$	194.1176

Malathion	121-75-5	Organophosphate	Insecticide, Acaricide, Veterinary substance	2.75	9.25	$[M+H]^+$	331.0433
Metalaxyl	57837-19-1	Phenylamide	Fungicide	1.75	7.71	$[M+H]^+$	280.1543
Methamidophos	10265-92-6	Organophosphate	Insecticide, Acaricide Metabolite	-0.82	1.06	$[M+H]^+$	142.0086
Methiathion	950-37-8	Organophosphate	Insecticide, Acaricide	2.57	8.54	$[M+H]^+$	302.9691
Methyl-Parathion	298-00-0	Organophosphate	Insecticide	3	8.93	$[M+H]^+$	264.0090
Metolachlor	51218-45-2	Chloroacetamide	Herbicide	3.4	9.44	/	/
Monocrotophos	6923-22-4	Organophosphate	Insecticide, Acaricide	1.45	4.53	$[M+H]^+$	224.0682
Myclobutanil	88671-89-0	Triazole	Fungicide	2.89	8.73	$[M+H]^+$	289.1215
Omethoate	1113-02-6	Organophosphate	Insecticide, Acaricide, Metabolite	-0.74	3.56	$[M+H]^+$	214.0297
Oxadixyl	77732-09-3	Phenylamide	Fungicide	0.65	6.71	$[M+H]^+$	279.1339
Parathion	56-38-2	Organophosphate	Insecticide, Acaricide	3.83	9.86	$[M+H]^+$	292.0403
Pendimethalin	40487-42-1	Dinitroaniline	Herbicide	5.4	11.21	$[M+H]^+$	282.1448
Phorate	298-02-2	Organophosphate	Insecticide, Acaricide, Nematicide	3.86	10.31	/	/
Phorate sulfone	251386	Organophosphate	Insecticide, Metabolite	4.14	8.38	/	/
Phorate-oxon sulfoxide	251417	Organophosphate	Insecticide, Metabolite	3.49	7.45	/	/
Phosalone	2310-17-0	Organophosphate	Insecticide, Acaricide	4.01	10.27	$[M+H]^+$	367.9941
Phosmet	732-11-6	Organophosphate	Insecticide, Acaricide,	2.8	8.7	$[M+H]^+$	318.0018

			Veterinary substance				
Phosphamidon	13171-21-6	Organophosphate	Insecticide, Acaricide	0.8	6.45	$[M+H]^+$	300.0762
Piperonyl butoxide	18693	Cyclic aromatic	Veterinary substance	4.75	10.88	$[M+H]^+$	334.1349
Pirimiphos methyl	29232-93-7	Organophosphate	Insecticide, Acaricide	4.2	10.27	$[M+H]^+$	306.1036
Profenofos	41198-08-7	Organophosphate	Insecticide, Acaricide	1.7	10.56	$[M+H]^+$	372.9424
Prometryn	7287-19-6	Triazine	Herbicide	3.34	7.61	$[M+H]^+$	242.1434
Propargite	2312-35-8	Sulphite ester	Acaricide	5.7	11.4	/	/
Propazine	139-40-2	Triazine	Herbicide	3.95	8.35	$[M+H]^+$	230.1167
Prothiofos	34643-46-4	Organophosphate	Insecticide	5.67	11.94	$[M+H]^+$	344.9701
Pyridaben	96489-71-3	Pyridazinone	Insecticide, Acaricide	6.37	11.75	$[M+H]^+$	365.1449
Pyridaphenthion	119-12-0	Organophosphate	Insecticide	3.2	8.93	$[M+H]^+$	341.0719
Pyrimethanil	53112-28-0	Anilinopyrimidine	Fungicide	2.84	7.45	$[M+H]^+$	200.1182
Pyroquilon	57369-32-1	Unclassified	Fungicide	1.57	6.33	$[M+H]^+$	174.0913
Quinalphos	13593-03-8	Organophosphate	Insecticide, Acaricide	4.44	9.79	$[M+H]^+$	299.0614
Tolclofos methyl	57018-04-9	Chlorophenyl	Fungicide	3.8	6.42	/	/
Tau-Fluvalinate	102851-06-9	Pyrethroid	Insecticide, Veterinary substance, Acaricide	7.02	11.98	$[M+H]^+$	503.1344
Triadimenol	55219-65-3	Triazole	Fungicide, Metabolite	3.18	8.3	/	/
Triazophos	24017-47-8	Organophosphate	Insecticide, Acaricide, Nematicide	3.55	9.27	$[M+H]^+$	314.0723
Triticonazole	131983-72-7	Triazole	Fungicide	3.29	8.5	/	/
Vamidothion	2275-23-2	Organophosphate	Insecticide, Acaricide	0.15	5.21	$[M+H]^+$	288.0488

Table S2 Latin plant names, botanical classifications, categories in the GB 23200.121-2021 National Food Safety Standard, categories in the Document Nº SANTE/11312/2021, and code information in mass-spectrometry-related and chromatography-related comparisons of 32 commodities.

**Name**	**Latin name**	**Family**	**Categories in the GB 2763-2021 National Food Safety Standard**	**Categories in the Document Nº SANTE/11312/2021**	**Codes in mass-spectrometry-related comparisons**
Cabbage	*Brassica oleracea* L.	Brassicaceae	Vegetables- Brasscia class-Ball brassical	High water content-Brassica vegetables	IDA8<br>MRM8
Mint	*Mentha haplocalyx* Briq.	Lamiaceae	Condiment-Leaf	High water content-Leafy vegetables and fresh herbs	IDA20<br>MRM20
Wheatgrass	*Agropyron cristatum* (L.) Gaertn.	Poaceae	Vegetable-Leaf Vegetable-Green leafy vegetables	High water content-Leafy vegetables and fresh herbs	IDA30<br>MRM30
Amomum tsao-ko	*Amomum tsaoko* Crevost et Lemarie	Zingiberaceae	Condiment-Fruits	High water content- Stem and stalk vegetables	IDA2<br>MRM2
Orange	*Citrus sinensis* (L.) Osbeck	Rutaceae	Fruits-Citurs	High acid content and high water content-Citrus fruit	IDA22<br>MRM22
Soybean	*Glycine max* (Linn.) Merr.	Fabaceae	Oil and grease- Large-scale oil seeds class	High oil content and very low water content-Oil seeds	IDA29<br>MRM29
Red chili	*Capsicum annuum* L.	Solanaceae	Vegetable- Solanaceous Vegetable- Other Solanaceous Vegetable	High water content-fruiting vegetables/cucurbits	IDA25<br>MRM25

Amaranth	*Amaranthus tricolor* L.	Amaranthaceae	Vegetable-Leaf Vegetable-Green leafy vegetables	High water content-Leafy vegetables and fresh herbs	IDA1<br>MRM1
Sichuan pepper	*Zanthoxylum bungeanum* Maxim	Rutaceae	Condiment-Fruits	/	IDA28<br>MRM28
Cowpea	*Vigna unguiculata* (Linn.) Walp.	Fabaceae	Vegetable-Legume- Pod edible legume	High water content-Fresh legume vegetables	IDA11<br>MRM11
Blueberry	*Vaccinium Spp.*	*Ericaceae*	Fruits- berries and other small fruit- bind and shrubs	High acid content and high water content-Small fruit and berries	IDA7<br>MRM7
Winged bean	*Psophocarpus tetragonolobus* (Linn.) D.C.	*Fabaceae*	Vegetable- Legume - Pod edible legume	High water content-Fresh legume vegetables	IDA31<br>MRM31
Artemisia selengensis	*Artemisia selengensis* Turcz. ex Bess.	*Asteraceae*	Vegetable- Leaf Vegetable-Green leafy vegetables	High water content-Stem and stalk vegetables	IDA3<br>MRM3
Asparagus	*Asparagus officinalis* L.	*Asparagaceae*	Vegetable-Stem	High water content-Stem and stalk vegetables	IDA4<br>MRM4
Basil	*Ocimum basilicum*	*Lamiaceae*	Condiment-Leaf	High water content-Leafy vegetables and fresh herbs	IDA5<br>MRM5

Green tea	/	/	Beverage	High water content-Difficult or unique commodities	IDA16<br>MRM16
Rosemary	*Rosmarinus officinalis*	*Lamiaceae*	Condiment-Leaf	High water content-Stem and stalk vegetables	IDA26<br>MRM26
Lemon	*Citrus limon* (L.) Burm. f.	*Rutaceae*	Fruits- Citurs	High acid content and high water content-Citrus fruit	IDA18<br>MRM18
Oyster mushroom	*Pleurotus ostreatus*	*Pleurotaceae*	Edible fungi- Mushrooms	High water content-Fresh Fungi	IDA23<br>MRM23
Green chili	*Capsicum annuum* L.	*Solanaceae*	Vegetable- Solanaceous Vegetable- Other Solanaceous Vegetable	High water content-fruiting vegetables/cucurbits	IDA14<br>MRM14
Okra	*Abelmoschus esculentus* (L.) Moench	*Malvaceae*	Vegetable- Solanaceous Vegetable- Other Solanaceous Vegetable	High water content-Fruiting vegetables/cucurbits	IDA21<br>MRM21
Chinese yam	*Dioscorea oppositifolia* L.	*Dioscoreaceae*	Vegetable- Tuber vegetable- Other tuber vegetable	High water content-Root and tuber vegetables	IDA9<br>MRM9
Ginger	*Zingiber officinale* Roscoe	*Zingiberaceae*	Vegetable- Rhizomes	High water content-Root and tuber vegetables	IDA13<br>MRM13

Garlic sprout	*Allium sativum* L.	*Liliaceae*	Vegetable- Stem brassicas	High water content-Stem and stalk vegetables	IDA12 MRM12
Pea seedlings	Pisum sativum L.	*Fabaceae*	Vegetable- Sprouts	High water content-Fresh legume vegetables	IDA24 MRM24
Zucchini	*Cucurbita pepo L.*	*Cucurbitaceae*	Vegetable- Gourd- Small gourd	High water content-fruiting vegetables/cucurbits	IDA32 MRM32
Maize	*Zea mays* L.	*Poaceae*	Creal- Dry grain	High starch and low water and fat content- Cereal grain and products thereof	IDA19 MRM19
Shiitake mushroom	*Lentinus edodes* (Berk.) Sing	*Omphalotaceae*	Edible fungi- Mushrooms	High water content-Fresh Fungi	IDA27 MRM27
Bay leaf	*Laurus nobilis* L.	*Lauraceae*	Condiment-Leaf	/	IDA6 MRM6
Cilantro	*Coriandrum sativum* L.	*Apiaceae*	Condiment-Leaf	High water content-Leafy vegetables and fresh herbs	IDA10 MRM10
Houttuynia cordata	*Herba Houttuyniae*	*Saururaceae*	Medicinal plant- Leaves and stem	High water content-Stem and stalk vegetables	IDA17 MRM17

Green Pimiento	*Capsicum frutescens* L. *(syn. C. annuum* L.） *var.grossum Bailey.*	*Solanaceae*	Vegetable- Solanaceous Vegetable- Other Solanaceous Vegetable	High water content-fruiting vegetables/cucurbits	IDA15<br>MRM15

Table S3 UHPLC-MS/MS ion transitions, declustering potential (DP), entrance potential (EP), collision energy (CE) and cell exit potential (CXP).

**Precusor ion (m/z)**	**Product ion (m/z)**	**ID**	**DP (V)**	**EP (V)**	**CE (eV)**	**CXP (V)**
270.0	238.1	Alachlor 1	41	10	17	14
270.0	162.2	Alachlor 2	41	10	27	10
270.0	146.9	Alachlor 3	41	10	41	18
228.1	68.0	Ametryn 1	156	10	59	8
228.1	96.0	Ametryn 2	156	10	39	26
228.1	91.0	Ametryn 3	156	10	35	10
215.9	174.2	Atrazine1	120	10	24	13
215.9	146.0	Atrazine 2	120	10	30	13
215.9	132.0	Atrazine 3	120	10	32	13
215.9	104.0	Atrazine 4	120	10	38	13
192.0	160.0	Carbendazim 1	41	10	25	10
192.0	132.0	Carbendazim 2	41	10	41	14
192.0	105.0	Carbendazim 3	41	10	49	16
192.0	117.0	Carbendazim 4	41	10	43	14
192.0	133.0	Carbendazim 5	41	10	41	12
192.0	106.1	Carbendazim 6	41	10	51	12
197.0	117.1	Chlordimeform 1	151	10	37	12
197.0	89.0	Chlordimeform 2	151	10	61	12
197.0	90.1	Chlordimeform 3	151	10	61	14
197.0	63.0	Chlordimeform 4	151	10	81	16
197.0	133.0	Chlordimeform 5	151	10	32	16
197.0	106.1	Chlordimeform 6	151	10	60	16
350.0	96.9	Chlorpyrifos 1	82	10	49	9
350.0	197.9	Chlorpyrifos 2	82	10	29	9
350.0	125.0	Chlorpyrifos 3	82	10	25	16

350.0	153.0	Chlorpyrifos 4	82	10	19	13
350.0	106.9	Chlorpyrifos 5	82	10	77	12
321.9	145.1	Chlorpyrifos methyl 1	56	10	19	10
321.9	85.1	Chlorpyrifos methyl 2	56	10	35	10
321.9	58.1	Chlorpyrifos methyl 3	56	10	57	8
359.1	167.1	Chlorsulforon 1	82	10	29	12
359.1	141.1	Chlorsulforon 2	82	10	23	14
359.1	142.2	Chlorsulforon 3	82	10	25	8
359.1	168.0	Chlorsulforon 4	82	10	25	10
362.9	227.0	Coumaphos 1	176	10	35	14
362.9	306.9	Coumaphos 2	176	10	25	18
362.9	335.0	Coumaphos 3	176	10	21	20
362.9	131.1	Coumaphos 4	176	10	73	16
413.0	295.0	Cyflufenamid 1	50	10	23	16
413.0	241.1	Cyflufenamid 2	50	10	31	14
413.0	359.3	Cyflufenamid 3	50	10	19	13
413.0	203.0	Cyflufenamid 4	50	10	57	12
413.0	341.2	Cyflufenamid 5	50	10	19	13
413.0	91.0	Cyflufenamid 6	50	10	71	10
192.1	119.1	DEET 1	91	10	25	6
192.1	91.0	DEET 2	91	10	41	14
192.1	65.1	DEET 3	91	10	63	10
192.1	72.1	DEET 4	91	10	29	8
305.0	169.0	Diazinon 1	120	10	27	11
305.0	153.0	Diazinon 2	120	10	28	11
220.9	109.1	Dichlorvos 1	136	10	23	8
220.9	79.0	Dichlorvos 2	136	10	39	12
220.9	95.0	Dichlorvos 3	136	10	49	12
220.9	60.1	Dichlorvos 4	136	10	75	8

268.1	226.0	Diethofencarb 1	31	10	15	14
268.1	123.9	Diethofencarb 2	31	10	45	14
268.1	180.1	Diethofencarb 3	31	10	25	12
268.1	152.1	Diethofencarb 4	31	10	31	10
406.1	337.0	Difenoconazole 1	120	10	23	7
406.1	251.0	Difenoconazole 2	120	10	35	16
406.1	188.0	Difenoconazole 3	120	10	61	10
406.1	75.0	Difenoconazole 4	120	10	115	10
406.1	111.0	Difenoconazole 5	120	10	73	12
276.0	244.0	Dimethenamid 1	80	10	21	18
276.0	168.1	Dimethenamid 2	80	10	33	10
276.0	111.0	Dimethenamid 3	80	10	43	14
276.0	126.0	Dimethenamid 4	80	10	47	16
230.0	199.0	Dimethoate 1	60	10	13	18
230.0	125.0	Dimethoate 2	60	10	29	14
230.0	171.0	Dimethoate 3	60	10	21	12
230.0	79.0	Dimethoate 4	60	10	45	36
328.0	283.0	Edifenphos 1	90	10	25	16
328.0	109.0	Edifenphos 2	90	10	49	16
328.0	311.3	Edifenphos 3	90	10	14	13
328.0	111.0	Edifenphos 4	90	10	35	14
328.0	173.1	Edifenphos 5	90	10	32	13
886.2	158.1	Emamectin Benzote 1	60	10	43	10
886.2	302.1	Emamectin Benzote 2	60	10	41	20
886.2	126	Emamectin Benzote 3	60	10	79	14
886.2	108.1	Emamectin Benzote 4	60	10	139	12
886.2	98.1	Emamectin Benzote 5	60	10	117	8
886.2	123.0	Emamectin Benzote 6	60	10	107	16
330.3	121.0	Epoxiconazole 1	120	10	27	9

330.3	101.1	Epoxiconazole 2	120	10	67	12
330.3	141.0	Epoxiconazole 3	120	10	23	13
330.3	75.0	Epoxiconazole 4	120	10	103	12
330.3	123.0	Epoxiconazole 5	120	10	23	13
330.3	74.0	Epoxiconazole 6	120	10	153	20
226.1	106.9	Ethiofencarb 1	61	10	21	9
226.1	164.1	Ethiofencarb 2	61	10	11	9
226.1	77.1	Ethiofencarb 3	120	10	59	12
384.9	199.0	Ethion 1	60	10	15	14
384.9	143.1	Ethion 2	60	10	37	12
384.9	97.0	Ethion 3	60	10	77	12
243.2	97.0	Ethoprophos 1	80	10	47	12
243.2	173.2	Ethoprophos 2	80	10	20	13
243.2	130.9	Ethoprophos 3	80	10	29	14
243.2	121.1	Ethoprophos 4	80	10	31	14
243.2	139.0	Ethoprophos 5	80	10	20	13
243.2	201.1	Ethoprophos 6	80	10	16	13
331.2	268.0	Fenarimol 1	161	10	31	16
331.2	111.0	Fenarimol 2	161	10	79	12
331.2	81.0	Fenarimol 3	161	10	51	10
320.9	146.0	Fenchlorphos 1	70	10	19	16
320.9	145.0	Fenchlorphos 2	70	10	19	8
320.9	85.0	Fenchlorphos 3	70	10	35	14
321.2	304.0	Fenchlorphos 4	70	10	10	13
278.1	125.0	Fenitrothion 1	120	10	28	13
278.1	67.1	Fenitrothion 2	120	10	49	18
278.1	108.1	Fenitrothion 3	120	10	37	8
278.1	109.0	Fenitrothion 4	120	10	43	12
208.1	152.0	Fenobucarb 1	65	10	13	9

208.1	95.0	Fenobucarb 2	65	10	19	10
350.3	97.2	Fenpropathrin 1	85	10	46	7
350.3	125.1	Fenpropathrin 2	50	10	25	13
279.0	169.1	Fenthion 1	56	10	25	12
279.1	246.8	Fenthion 2	120	10	18	13
384.0	282.1	Fluazifop butyl 1	161	10	29	18
384.0	328.1	Fluazifop butyl 2	161	10	23	22
384.0	254.0	Fluazifop butyl 3	161	10	39	14
384.0	91.0	Fluazifop butyl 4	161	10	43	12
266.2	228.9	Fludioxonil 1	66	10	23	14
266.2	158.0	Fludioxonil 2	66	10	47	16
266.2	185.1	Fludioxonil 3	66	10	35	12
376.1	306.8	Fluquinconazole 1	176	10	37	18
376.1	349.0	Fluquinconazole 2	176	10	29	22
376.1	108.0	Fluquinconazole 3	176	10	75	14
302.0	70.0	Flutriafol 1	71	10	21	10
302.0	123.0	Flutriafol 2	71	10	39	14
302.0	95.0	Flutriafol 3	71	10	73	12
302.0	75.0	Flutriafol 4	71	10	101	10
353.1	228.0	Hexythiazox 1	36	10	23	14
353.1	168.1	Hexythiazox 2	36	10	35	10
289.0	91.0	Iprobenfos 1	51	10	39	14
289.0	205.0	Iprobenfos 2	51	10	15	14
289.0	247.0	Iprobenfos 3	51	10	11	16
289.0	65.1	Iprobenfos 4	51	10	83	10
314.0	162.1	Isazofos 1	186	10	25	10
314.0	97.0	Isazofos 2	186	10	55	12
314.0	120.0	Isazofos 3	70	10	40	9
314.0	119.1	Isazofos 4	186	10	49	6

211.0	152.1	Isoprocarb 1	41	10	17	14
211.0	95.0	Isoprocarb 2	25	10	24	7
331.1	127.1	Malathion 1	90	10	19	8
331.1	285.0	Malathion 2	90	10	11	20
331.1	99.0	Malathion 3	90	10	33	12
331.1	125.0	Malathion 4	90	10	41	12
278.0	210.0	Metazachlor 1	50	10	15	13
278.0	134.0	Metazachlor 2	50	10	30	13
278.0	105.1	Metazachlor 3	31	10	53	12
278.0	77.1	Metazachlor 4	31	10	81	14
280.1	220.1	Metalaxyl 1	56	10	19	14
280.1	160.2	Metalaxyl 2	56	10	33	10
280.2	192.3	Metalaxyl 3	56	10	24	11
141.9	94.1	Methamidophos 1	56	10	23	12
141.9	125.0	Methamidophos 2	54	10	18	9
141.9	64.0	Methamidophos 3	56	10	35	10
303.1	145.1	Methiathion 1	121	10	15	10
303.1	85.1	Methiathion 2	121	10	31	38
264.0	232.0	Methyl Parathion 1	46	10	23	16
264.0	75.1	Methyl Parathion 2	46	10	59	12
284.1	252.1	Metolachlor 1	56	10	21	14
284.1	176.2	Metolachlor 2	56	10	35	12
284.1	91.1	Metolachlor 3	56	10	69	10
284.1	77.1	Metolachlor 4	56	10	97	12
224.0	127.0	Monocrotophos 1	126	10	23	16
224.0	98.1	Monocrotophos 2	126	10	19	12
224.0	58.0	Monocrotophos 3	126	10	41	16
224.0	109.1	Monocrotophos 4	126	10	43	14
289.0	70.0	Myclobutanil 1	136	10	23	8

289.0	125.0	Myclobutanil 2	136	10	49	14
289.0	89.0	Myclobutanil 3	136	10	83	10
214.0	125.1	Omethoate 1	111	10	29	4
214.0	155.0	Omethoate 2	111	10	21	10
214.0	109.0	Omethoate 3	111	10	37	16
214.0	182.9	Omethoate 4	56	10	16	6
279.1	219.1	Oxadixyl 1	71	10	17	14
279.1	132.2	Oxadixyl 2	71	10	45	8
279.1	101.9	Oxadixyl 3	100	10	15	13
279.1	117.0	Oxadixyl 4	71	10	69	14
292.2	96.9	Parathion 1	61	10	53	38
292.2	264.0	Parathion 2	60	10	15	13
282.1	212.0	Pendimethalin 1	36	10	15	12
282.1	194.1	Pendimethalin 2	36	10	25	16
408.0	153.1	Permethrin 1	76	10	65	10
261.0	75.0	Phorate 1	51	10	23	10
261.0	96.9	Phorate 2	51	10	39	14
261.0	143.0	Phorate 3	51	10	25	8
261.0	199.0	Phorate 4	51	10	10	10
293.0	247.0	Phorate sulfone 1	120	10	10	13
293.0	171.0	Phorate sulfone 2	120	10	16	13
276.9	171.0	Phorate-oxon sulfoxide 1	80	10	17	13
276.9	199.0	Phorate-oxon sulfoxide 2	56	10	15	12
276.9	153.1	Phorate-oxon sulfoxide 3	80	10	20	13
276.9	97.0	Phorate-oxon sulfoxide 4	56	10	47	12
276.9	143.0	Phorate-oxon sulfoxide 5	56	10	29	14
276.9	78.9	Phorate-oxon sulfoxide 6	56	10	81	10
367.9	182.0	Phosalone 1	56	10	25	12
367.9	111.1	Phosalone 2	56	10	57	10

367.9	75.1	Phosalone 3	56	10	97	12
367.9	74.1	Phosalone 4	56	10	117	8
367.9	322.0	Phosalone 5	120	10	13	13
318.1	256.3	Phosmet 1	60	10	31	16
318.1	160.0	Phosmet 2	60	10	19	30
318.1	77.0	Phosmet 3	60	10	75	8
300.0	174.1	Phosphamidon 1	156	10	19	8
300.0	127.0	Phosphamidon 2	156	10	31	16
300.0	72.1	Phosphamidon 3	156	10	47	8
300.0	108.9	Phosphamidon 4	156	10	63	12
356.2	177.1	Piperonyl butoxide 1	81	10	19	18
356.2	119.1	Piperonyl butoxide 2	81	10	49	14
356.2	147.0	Piperonyl butoxide 3	100	10	45	13
356.2	91.0	Piperonyl butoxide 4	81	10	75	26
356.2	149.0	Piperonyl butoxide 5	81	10	47	18
306.0	164.1	Pirimiphos methyl 1	156	10	29	10
306.0	108.0	Pirimiphos methyl 2	156	10	41	12
306.0	67.0	Pirimiphos methyl 3	156	10	59	8
306.0	125.0	Pirimiphos methyl 4	156	10	43	10
334.0	198.1	Primiphos ethyl 1	6	10	31	12
334.0	182.1	Primiphos ethyl 2	6	10	31	12
334.0	170.1	Primiphos ethyl 3	6	10	43	10
334.0	96.9	Primiphos ethyl 4	6	10	65	12
372.9	302.7	Profenofos 1	156	10	27	22
372.9	344.9	Profenofos 2	156	10	19	22
372.9	128.0	Profenofos 3	156	10	55	16
372.9	97.0	Profenofos 4	156	10	41	16
242.1	158.1	Prometryne 1	221	10	33	10
242.1	200.1	Prometryne 2	221	10	27	12

242.1	85.0	Prometryne 3	221	10	43	10
242.1	68.1	Prometryne 4	221	10	99	4
242	116.1	Prometryne 5	166	10	39	14
368.1	231.2	Propargite 1	36	10	15	16
368.1	175.2	Propargite 2	36	10	23	4
230.0	188.0	Propazine 1	80	10	25	12
230.0	146.1	Propazine 2	80	10	33	10
230.0	104.0	Propazine 3	80	10	43	12
230	78.9	Propazine 4	80	10	45	12
344.8	240.7	Prothiofos 1	80	10	25	16
344.8	268.9	Prothiofos 2	80	10	17	20
344.8	132.9	Prothiofos 3	80	10	71	16
344.8	160.9	Prothiofos 4	80	10	47	12
344.8	176.9	Prothiofos 5	80	10	41	10
344.8	161.9	Prothiofos 6	80	10	55	12
365.0	308.9	Pyridaben 1	80	10	19	20
365.0	147.1	Pyridaben 2	80	10	35	10
365.0	117.0	Pyridaben 3	80	10	87	14
365.0	98.0	Pyridaben 4	80	10	61	12
341.0	189.1	Pyridaphenthion 1	80	10	31	12
341.0	92.1	Pyridaphenthion 2	80	10	53	10
341.0	65.0	Pyridaphenthion 3	80	10	75	8
341.0	97.0	Pyridaphenthion 4	80	10	73	14
200.0	107.0	Pyrimethanil 1	211	10	33	16
200.0	77.0	Pyrimethanil 2	211	10	55	10
200.0	67.1	Pyrimethanil 3	211	10	57	8
200.0	51.0	Pyrimethanil 4	211	10	91	24
174.0	132.1	Pyroquilon 1	166	10	33	10
174.0	117.1	Pyroquilon 2	166	10	45	12

174.0	89.0	Pyroquilon 3	166	10	75	12
174.0	77.0	Pyroquilon 4	166	10	67	10
299.0	97.0	Quinalphos 1	80	10	49	12
299.0	163.1	Quinalphos 2	80	10	31	10
299.0	147.1	Quinalphos 3	80	10	31	10
299.0	119.1	Quinalphos 4	80	10	59	14
503.0	208.1	Tau Fluvalinate 1	80	10	19	14
503.0	181.1	Tau Fluvalinate 2	80	10	45	20
503.0	152.0	Tau Fluvalinate 3	80	10	99	8
503.0	295.1	Tau Fluvalinate 4	80	10	17	13
258.1	125.0	Thiobencarb 1	80	10	25	8
258.2	57.0	Thiobencarb 2	80	10	47	8
296.1	70.0	Triadimenol 1	80	10	39	10
296.1	149.0	Triadimenol 2	80	10	27	10
314	162.1	Triazophos-1	80	10	25	10
314	119.6	Triazophos-2	80	10	49	10
314	120	Triazophos-3	80	10	35	10
314	97	Triazophos-4	80	10	57	10
318.2	70.0	Triticonazole 1	80	10	55	10
318.2	125.1	Triticonazole 2	80	10	31	13
288.0	146.2	Vamidothion 1	80	10	17	10
288.0	118.0	Vamidothion 2	80	10	35	14
288.0	58.0	Vamidothion 3	80	10	53	8
288.0	86.1	Vamidothion 4	80	10	33	10

Table S4. Matrix species with pesticides with all their MRM transitions showing MEs > 100% under MRM scan mode.

Pesticides	Matrix
Carbendazim	Basil, Cowpea, Green chili, Maize
Difenoconazole	Basil, Cowpea
Methiathion	Amaranth, Basil, Blueberry, Cabbage, Chinese yam, Green tea, Green chili, Lemon, Mint, Pea seedling, Red chili, Soybean
Myclobutanil	Lemon
Propargite	Amaranth, *Aritemisia selengensis*, Asparagus, Basil, Blueberry, Cabbage, Cowpea, Garlic sprout, Green tea, Mint, Okra, Oyster mushroom, Red chili, Shiitake mushroom, Soybean, Green pimiento
Tau-fluvalinate	Soybean

Table S5. List of 20 pesticides with all their 69 MRM transitions as differential pesticide MRM transitions between groups G1 and G2.

Chemical classes	Pesticides
Amidoxine	Cyflufenamid
Aryloxyphenoxypr-opionate	Fluazifop-butyl
Carboxamide	Hexythiazox
Chloroaceta mide	Dimethenamid, Alachlor,
Organophosphate	Chlorpyrifos, Coumaphos, Diazinon, Dimethoate, Ethion, Isazofos, Parathion, Pirimiphos-methyls, Prothiofos, Quinalphos
Sulfonylurea	Chlorsulforon
Triazine	Propazine
Triazole	Triadimenol, Triconazole

Table S6. 13 positive pesticides in corresponding matrices in MS/MS scan under IDA mode by UPLC QTOF

Pesticide Name	Matrices	MRLs (mg/kg)	Addition level (mg/kg)	S/N	Found at mass (Da)	Error (ppm)	Found at t_R (min)	Library hit	Library score
Chlorpyrifos	Asparagus	0.05	0.05	581.8	321.9027	1.3	11.15	Chlorpyrifos	97.7
Chlorpyrifos	Orange	2	0.05	682.1	321.9028	1.6	11.07	Chlorpyrifos	97.7
Chlorpyrifos	Lemon	2	0.05	707.6	321.9027	1.4	11.13	Chlorpyrifos	98.2
Chlorpyrifos	Green tea	2	0.625	1196	321.903	2.4	11.12	Chlorpyrifos	94.7
Chlorpyrifos	Mint	2	0.625	686.8	321.9031	2.7	11.13	Chlorpyrifos	97.4
Chlorpyrifos	*Amomum tsao-ko*	2	0.625	355.8	321.9029	1.9	11.12	/	/
Chlorpyrifos	Sichuan pepper	2	0.625	233.3	321.902	0.9	11.11	/	/
Coumaphos	Orange	0.05	0.05	2986.5	363.0022	1.3	10.12	Coumaphos	100
Coumaphos	Blueberry	0.05	0.05	2028.3	363.0222	1.4	10.11	Coumaphos	99.8
Coumaphos	Lemon	0.05	0.05	1131.3	363.0222	1.2	10.09	Coumaphos	99.7
Coumaphos	Cabbage	0.05	0.05	814.5	363.0225	2.2	10.11	Coumaphos	99.6
Coumaphos	Wheat grass	0.05	0.05	1563	363.0223	1.5	10.13	Coumaphos	100
Coumaphos	Red chili	0.05	0.05	1701.4	363.0227	2.7	10.15	Coumaphos	99.5
Coumaphos	Amaranth	0.05	0.05	2390.6	363.0223	1.5	10.17	Coumaphos	99.2
Coumaphos	Cowpea	0.05	0.05	1178.4	363.0226	2.3	10.11	Coumaphos	100
Coumaphos	Winged bean	0.05	0.05	40716.4	363.0221	1.1	10.11	Coumaphos	99.3
Coumaphos	*Artemisia selengensis*	0.05	0.05	2123.9	363.0223	1.5	10.15	Coumaphos	99.6
Coumaphos	Asparagus	0.05	0.05	3544.4	363.0221	1	10.18	Coumaphos	99.5
Coumaphos	Green chili	0.05	0.05	40260.7	363.0222	1.3	10.1	Coumaphos	99.7
Coumaphos	Okra	0.05	0.05	53195.1	363.0226	2.5	10.11	Coumaphos	99.6
Coumaphos	Chinese yam	0.05	0.05	41005.6	363.0223	1.5	10.12	Coumaphos	99.6

Coumaphos	Ginger	0.05	0.05	20611.8	363.0231	3.8	10.11	Coumaphos	98.8
Coumaphos	Garlic sprout	0.05	0.05	601	363.0219	0.5	10.11	Coumaphos	100
Coumaphos	Pea seedling	0.05	0.05	996	363.0224	1.7	10.11	Coumaphos	100
Coumaphos	Zucchini	0.05	0.05	834.4	363.0222	1.2	10.11	Coumaphos	100
Coumaphos	Green pimiento	0.05	0.05	429979	363.0223	1.4	10.11	Coumaphos	100
Dichlorvos	Orange	0.2	0.05	773.7	220.9534	0.9	6.88	Dichlorvos	96.7
Dichlorvos	Blueberry	0.2	0.05	1733.5	220.9535	1.3	6.87	Dichlorvos	93.9
Dichlorvos	Lemon	0.2	0.05	1770.5	220.9533	0.6	6.89	Dichlorvos	98.7
Dichlorvos	Cabage	0.5	0.05	2019.8	220.9538	2.3	6.88	Dichlorvos	98.5
Dichlorvos	Wheatgrass	0.2	0.05	1596.4	220.9533	0.6	6.85	Dichlorvos	99.1
Dichlorvos	Red chili	0.2	0.05	1544.7	220.953	1.5	6.85	Dichlorvos	97.6
Dichlorvos	Amaranth	0.2	0.05	1966.6	220.9536	1.8	6.89	Dichlorvos	98.3
Dichlorvos	Cowpea	0.2	0.05	1775.7	220.9535	1.5	6.82	Dichlorvos	99.2
Dichlorvos	Winged bean	0.2	0.05	2006.7	220.9534	0.9	6.83	Dichlorvos	96.5
Dichlorvos	*Artemisia selengensis*	0.2	0.05	1706.1	220.9536	1.8	6.84	/	/
Dichlorvos	Asparagus	0.2	0.05	2065.1	220.9534	1	6.84	Dichlorvos	98.3
Dichlorvos	Green chili	0.2	0.05	1895.2	220.9533	0.5	6.84	Dichlorvos	99.2
Dichlorvos	Okra	0.2	0.05	2079.1	220.9535	1.6	6.85	Dichlorvos	99.1
Dichlorvos	Chinese yam	0.2	0.05	1940.9	220.9532	0.1	6.89	Dichlorvos	100
Dichlorvos	Ginger	0.2	0.05	1183.8	220.9536	1.9	6.85	Dichlorvos	88.7
Dichlorvos	Garlic sprout	0.2	0.05	671.8	220.9535	1.4	6.82	Dichlorvos	98.9
Dichlorvos	Pea seedling	0.2	0.05	1510.6	220.953	1.4	6.82	Dichlorvos	98.5
Dichlorvos	Zucchini	0.2	0.05	786.2	220.9533	0.6	6.82	Dichlorvos	95.5
Dichlorvos	Green pimiento	0.2	0.05	1821.8	220.9535	1.5	6.81	Dichlorvos	98.1

Epoxiconazole	Orange	1	0.05	4069.3	330.0802	-0.5	8.82	Epoxiconazole	97.7
Fenarimol	Green pimiento	0.5	0.05	2166.6	331.0407	2.4	8.65	Fenarimol	99.9
Fenthion	Orange	0.05	0.05	2713.7	279.0279	0.9	9.85	Fenthion	97.7
Fenthion	Blueberry	0.05	0.05	3671.8	279.0275	0.7	9.86	Fenthion	99.7
Fenthion	Lemon	0.05	0.05	1986.1	279.0275	0.9	9.85	Fenthion	97.8
Fenthion	Cabbage	2	0.05	2213.6	279.0277	1.4	9.85	Fenthion	99.1
Fenthion	Wheat grass	0.05	0.05	6671.8	279.027	1.2	9.88	Fenthion	99.7
Fenthion	Red chili	0.05	0.05	3108	279.0278	1.8	9.85	Fenthion	99.5
Fenthion	Amaranth	0.05	0.05	3069.3	279.028	2.5	9.85	Fenthion	97.8
Fenthion	Cowpea	0.05	0.05	2250.2	279.0278	1.9	9.85	Fenthion	100
Fenthion	Winged bean	0.05	0.05	1819.2	279.0277	1.4	9.85	Fenthion	99.3
Fenthion	*Artemisia selengensis*	0.05	0.05	1166.9	279.0277	1.6	9.82	Fenthion	98.6
Fenthion	Asparagus	0.05	0.05	3722.9	279.0276	1.1	9.82	Fenthion	96.1
Fenthion	Green chili	0.05	0.05	4100.6	279.0277	1.4	9.87	Fenthion	98.3
Fenthion	Okra	0.05	0.05	7805.3	279.0279	2.2	9.87	Fenthion	98.7
Fenthion	Chinese yam	0.05	0.05	2622.5	279.027	1.1	9.88	Fenthion	97.9
Fenthion	Ginger	0.05	0.05	4560	279.0279	2.2	9.82	Fenthion	93.4
Fenthion	Garlic sprout	0.05	0.05	1270.5	279.0276	1.2	9.82	Fenthion	99.2
Fenthion	Pea seedling	0.05	0.05	2250.2	279.0278	1.9	9.82	Fenthion	100
Fenthion	Zucchini	0.05	0.05	2151.9	279.0275	0.9	9.83	Fenthion	99.2
Fenthion	Green pimiento	0.05	0.05	3340.7	279.0276	1	9.82	Fenthion	99.5
Hexythiazox	Orange	0.5	0.05	744.7	353.1089	1.2	11.25	Hexythiazox	25.8
Hexythiazox	Lemon	0.5	0.05	1922.7	353.1088	0.8	11.26	Hexythiazox	24.9
Hexythiazox	Green tea	15	0.625	2403.4	353.109	1.5	11.25	Hexythiazox	12.2

Methidathion	Orange	0.05	0.05	2806.7	302.9709	5.8	8.53	/	/
Methidathion	Blueberry	0.05	0.05	4649.8	302.9696	1.7	8.5	Methidathion	99
Methidathion	Lemon	0.05	0.05	3563.7	302.9689	-0.7	8.5	/	/
Methidathion	Cabbage	0.05	0.05	4112.9	302.9702	3.5	8.54	/	/
Methidathion	Wheat grass	0.05	0.05	5094.3	302.969	2.8	8.5	/	/
Methidathion	Red chili	0.05	0.05	4219.6	302.97	2.8	8.59	Methidathion	100
Methidathion	Amaranth	0.05	0.05	3392	302.9702	3.5	8.59	Methidathion	98.3
Methidathion	Cowpea	0.05	0.05	3398.8	302.9698	2.3	8.59	/	/
Methidathion	Winged bean	0.05	0.05	4415.7	302.9692	0.3	8.59	/	/
Methidathion	*Artemisia selengensis*	0.05	0.05	1722.6	302.9713	7.3	8.58	/	/
Methidathion	Asparagus	0.05	0.05	3820.3	302.9698	2.2	8.54	/	/
Methidathion	Green chili	0.05	0.05	4073	302.9698	2.4	8.55	Methidathion	100
Methidathion	Okra	0.05	0.05	3543.8	302.9701	3.3	8.55	/	/
Methidathion	Chinese yam	0.05	0.05	4065.7	302.9697	1.9	8.55	Methidathion	100
Methidathion	Ginger	0.05	0.05	1378.4	302.9705	4.6	8.53	/	/
Methidathion	Garlic sprout	0.05	0.05	82.8	302.9697	1.9	8.53	/	/
Methidathion	Pea seedling	0.05	0.05	3253.5	32.9699	2.5	8.52	/	/
Methidathion	Zucchini	0.05	0.05	122.1	302.9696	1.6	8.58	/	/
Methidathion	Green pimiento	0.05	0.05	3464	302.9697	1.9	8.58	Methidathion	96.9
Pendimethalin	Cabbage	0.5	0.05	814.5	363.0225	2.2	11.19	Pendimethalin	99.6
Pendimethalin	Asparagus	0.1	0.05	3544.4	363.0221	1	11.2	Pendimethalin	99.5
Phosalone	*Amomum tsao-ko*	2	0.625	204931	367.9951	2.6	10.25	/	/
Phosalone	Sichuan pepper	2	0.625	9811.5	367.9949	2.1	10.28	/	/
Phosmet	Orange	5	0.05	377.2	318.002	0.6	8.71	Phosmet	98.6

Phosmet	Lemon	5	0.05	443.4	318.0026	2.4	8.75	Phosmet	98.4
Phosmet	Blueberry	10	0.05	401.3	318.0025	2	8.75	Phosmet	97.3
Phosmet	Cabbage	0.5	0.05	135.7	318.0028	3	8.7	Phosmet	85.7
Profenofos	Cabbage	0.05	0.05	56759.2	372.9434	2.7	10.57	Profenofos	99.2
Profenofos	Red chili	3	0.05	90977.7	372.9431	1.9	10.57	Profenofos	99.2
Profenofos	Green chili	3	0.05	84789.2	372.9429	1.3	10.57	Profenofos	100
Profenofos	Orange	0.2	0.05	94685.3	372.943	1.7	10.6	Profenofos	99.5
Tau-fluvalinate	Cabbage	0.5	0.05	533.6	503.1036	2.3	11.99	/	/

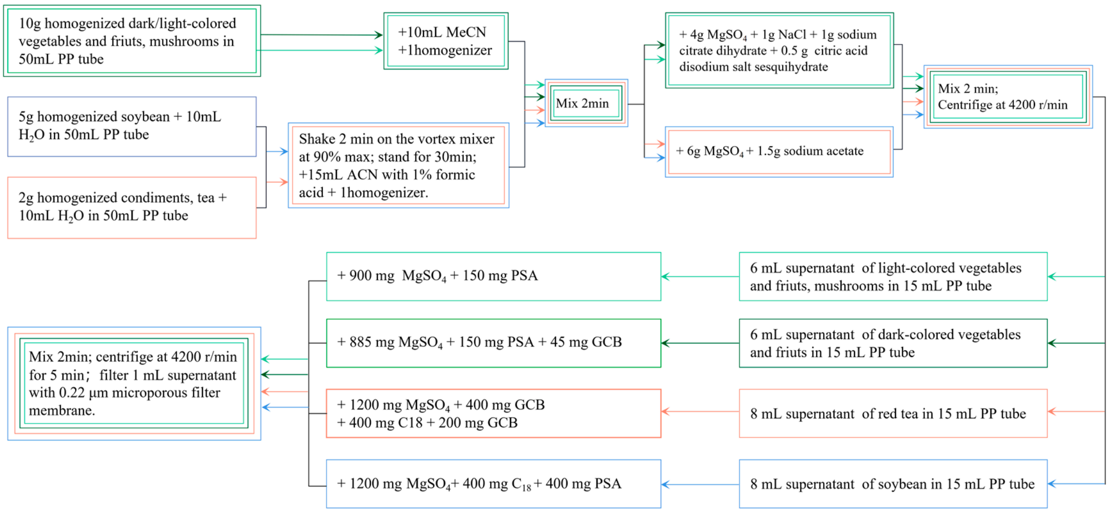

Figure S1. Sample preparation procedures according to the GB 23200.121-2021 National Food Safety Standard. The flow chart of the QuEChERS cleanup procedure for light fruits and vegetables and mushrooms were colored in light green, for dark fruits and vegetables was colored in dark green, for condiments and tea were colored in orange, and for soybeans were colored in blue.

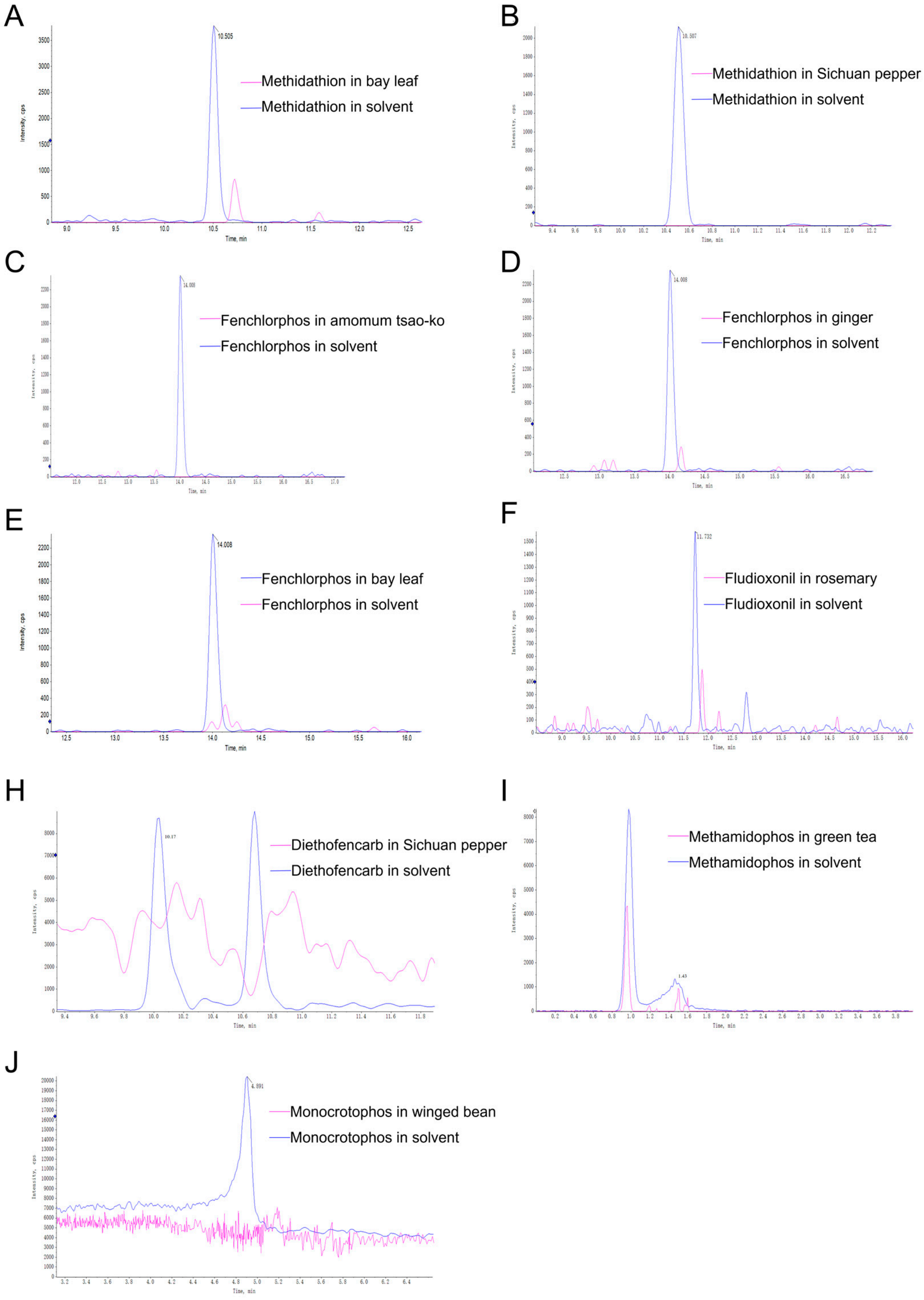

Figure S2. Extracted ion chromatogram (XIC) of pesticides with 100% signal suppression （MEs = -100%） in matrix-matched and reagent-only standard solutions.

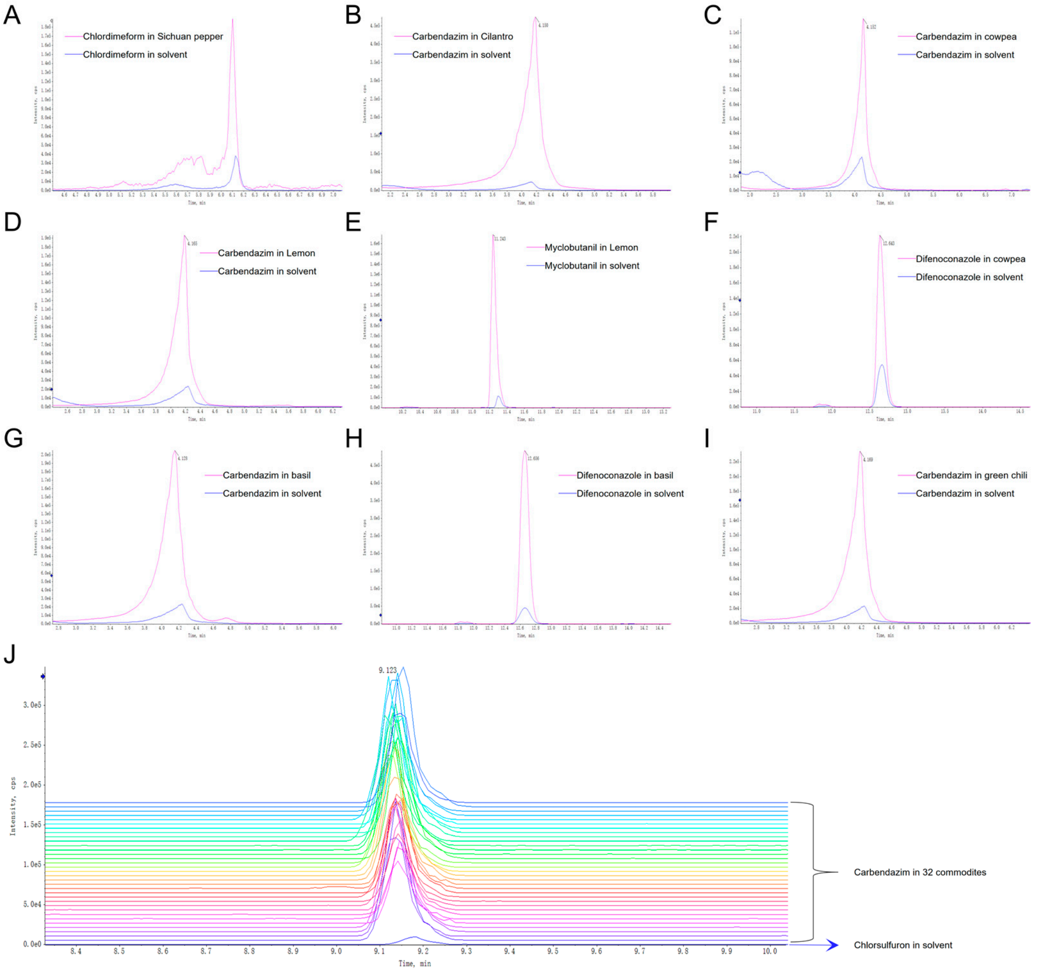

Figure S3. Extracted ion chromatogram (XIC) of pesticides with extreme signal enhancement (MEs > 100%) in matrix-matched and reagent-only standard solutions.

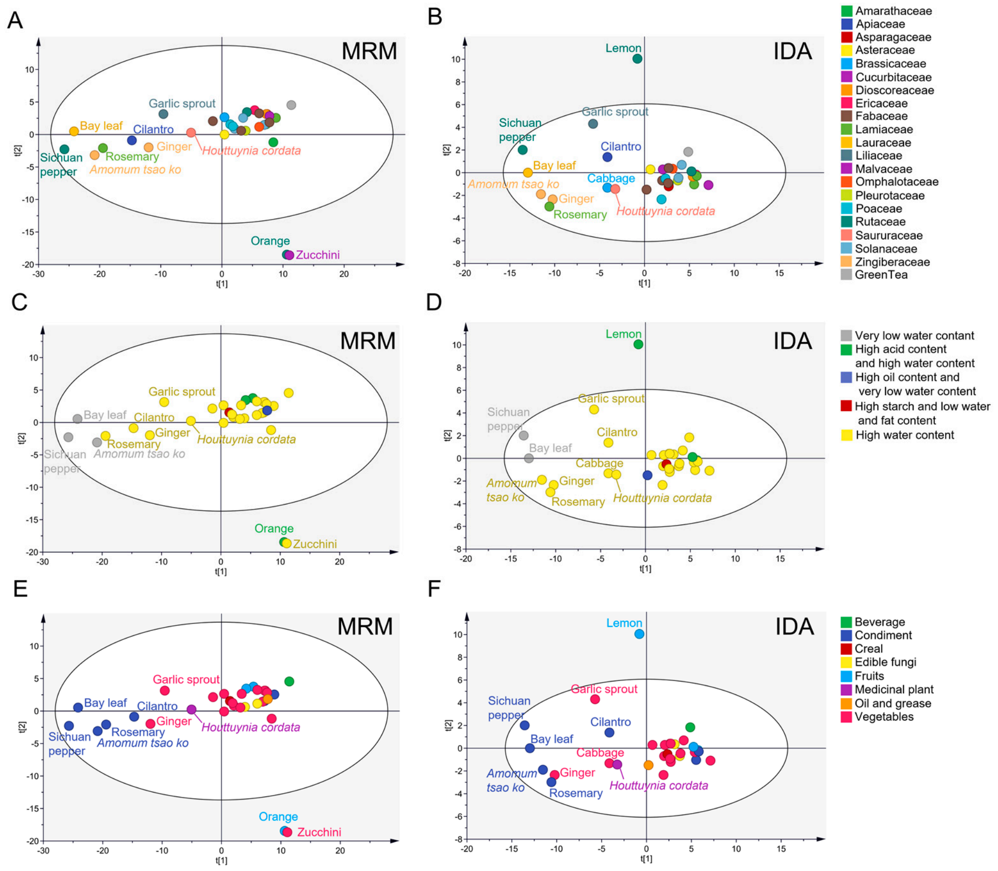

Figure S4. The score plots of PCA modeling under MRM and in TOF-MS scan under IDA mode, colored by botanical classifications (A) and (B), the Document Nº SANTE/11312/2021 (C) and (D), and the GB 2763-2021 National Food Safety Standard (E) and (F), respectively.

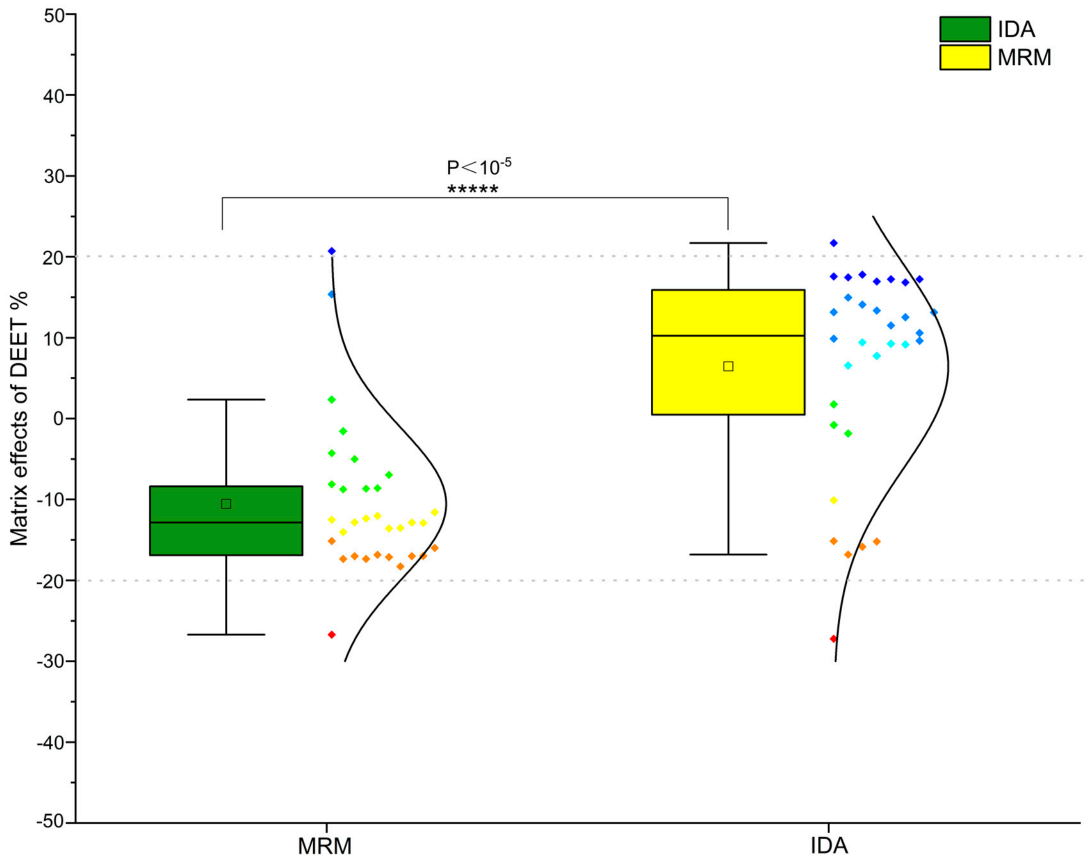

Figure S5. Distribution of DEET's MEs under IDA scan and MRM scan.
